# Identifying patients with less potential to benefit from implantable cardioverter-defibrillator therapy: comparison of the performance of four risk scoring systems

**DOI:** 10.1007/s10840-017-0243-9

**Published:** 2017-04-06

**Authors:** Amit Kaura, Nicholas Sunderland, Ravi Kamdar, Edward Petzer, Theresa McDonagh, Francis Murgatroyd, Para Dhillon, Paul Scott

**Affiliations:** 1 King’s College Hospital, Denmark Hill, London, SE5 9RS UK; 2 Hammersmith Hospital, Du Cane Road, London, W12 0HS UK

**Keywords:** Implantable cardioverter-defibrillator, Mortality, Scoring system, Urea

## Abstract

**Purpose:**

Patients at high non-sudden cardiac death risk may gain no significant benefit from implantable cardioverter-defibrillator (ICD) therapy. A number of approaches have been proposed to identify these patients, including single clinical markers and more complex scoring systems. The aims of this study were to use the proposed scoring systems to (1) establish how many current ICD recipients may be too high risk to derive significant benefit from ICD therapy and (2) evaluate how well the scoring systems predict short-term mortality in an unselected ICD cohort.

**Methods:**

We performed a single-centre retrospective observational study of all new ICD implants over 5 years (2009–2013). We used four published scoring systems (Bilchick, Goldenberg, Kramer and Parkash) and serum urea to identify new ICD recipients whose short-term predicted mortality risk was high. We evaluated how well the scoring systems predicted death.

**Results:**

Over 5 years, there were 406 new implants (79% male, mean age 70 (60–76), 58% primary prevention). During a follow-up of 936 ± 560 days, 96 patients died. Using the scoring systems, the proportion of ICD recipients predicted to be at high short-term mortality risk were 5.9% (Bilchick), 34.7% (Goldenberg), 7.4% (Kramer), 21.4% (Parkash) and 25% (urea, cut-off of >9.28 mM). All four risk scores predicted mortality (*P* < 0.0001); however, none outperformed urea for the prediction of 1- or 3-year mortality.

**Conclusions:**

Using published scoring systems, a significant proportion of current ICD recipients are at high short-term mortality risk. Although all four scoring systems predicted mortality during follow-up, none significantly outperformed serum urea.

## Introduction

The implantable cardioverter-defibrillator (ICD) is a highly effective therapy for the prevention of sudden cardiac death (SCD) in high-risk patients [1]. However, many patients whose risk of short-term mortality following device implantation is high may gain no significant benefit from an ICD, irrespective of their SCD risk. Such patients, who have significant non-cardiac comorbidity or advanced heart failure, typically die of a non-cardiac cause or pump failure [2, 3]. These patients are important as they are exposed to all of the risks of ICD therapy, without the opportunity to gain significant mortality benefit.

A number of complex scoring systems have been proposed to identify these high-risk patients [4–7] (Table 1). However, it is unclear which scoring system is most useful and whether any add incremental value compared to a single risk marker alone (serum urea).

**Table 1:** Scoring systems to identify patients at high risk of early mortality after ICD implantation

Clinical parameter	Scoring system			
	Goldenberg	Bilchick	Kramer	Parkash
Age	>70 years	≥75 years	≥70 years	>80 years
*Points*	*1*	*62*	*1*	*1*
Atrial fibrillation	✓	✓		✓
*Points*	*1*	*27*		*1*
Chronic kidney disease	Urea >9.28 mmol/L	✓	Creatinine ≥177 μmol/L	Creatinine >159 μmol/L
*Points*	*1*	*100*	*2*	*1*
COPD		✓		
*Points*		*62*		
Diabetes mellitus		✓		
*Points*		*41*		
LVEF		≤20%	≤20%	
*Points*		*28*	*1*	
NYHA functional class	>II	> II		>II
* Points*	*1*	*36*		*1*
Peripheral arterial disease			✓	
*Points*			*1*	
QRS duration	>120 ms			
*Points*	*1*			

*COPD* chronic obstructive pulmonary disease, *LVEF* left ventricular ejection fraction, *NYHA* New York Heart Association

The aims of this study were to use the proposed scoring systems to (1) establish how many current ICD recipients may be too high risk to derive significant benefit from ICD therapy and (2) evaluate how well the proposed scoring systems predict short-term mortality in an unselected cohort of ICD recipients.

## Methods

### Study design

We conducted a single-centre retrospective analysis of consecutive patients undergoing first time ICD implantation at King’s College Hospital (London, UK) between January 2009 and October 2013.

### Derivation of risk scores

The presence or absence of specific clinical variables such as atrial fibrillation (AF), diabetes, peripheral arterial disease (PAD) and chronic obstructive pulmonary disease at the time of ICD implant were determined by review of the clinical records.

Assigned or measured variables such as age, New York Heart Association (NYHA) heart failure functional class, creatinine, urea, QRS duration and left ventricular ejection fraction (LVEF) were taken at the time of ICD implant or the closest available value.

AF was defined as a history of paroxysmal or permanent AF on the electrocardiogram. PAD was defined as in the Kramer study; a patient had an intervention on the carotid arteries or lower extremities, thoracic or abdominal aorta or had clinical claudication [6]. Chronic kidney disease was defined as an estimated glomerular filtration rate of <60 mL/min/1.73m^2^ using the modification of diet in renal disease equation.

### Statistical analysis

Continuous variables are presented as median (interquartile range) and categorical variables are expressed as absolute and relative frequency.

The Bilchick [4], Goldenberg [5], Kramer [6] and Parkash [7] risk scores were calculated from patients’ clinical characteristics according to the original publications. On the basis of these risk scores, patients were further classified into risk categories as set out in the papers [4–7]. The Bilchick, Kramer and Parkash models distinguished two risk categories for mortality (low and high risk). In the Goldenberg model, patients were stratified in three risk categories for mortality (low, intermediate and high risk). For the purposes of our study, we combined the low and intermediate categories into one ‘low-risk’ group. We used serum urea to categorize patients into low and high risk, based on the value derived from the Goldenberg study, an analysis of MADIT-2 (cut-off of >9.28 mM) [5].

Cox proportional hazards regression modelling was used to evaluate the independent contribution of each of the clinical parameters within all scoring systems to the occurrence of mortality during follow-up. Each clinical parameter and risk scoring system was first entered into a univariate model, and those found to be significant at a level of *P* < 0.02 were then entered into a stepwise forward multivariate model.

Risk model calibration was assessed by the Hosmer-Lemeshow goodness-of-fit test, which determines how close the predicted and observed incidence of events is over a range of scores. In this test, a significant result indicates lack of model adjustment.

We assessed the discriminatory capacity of the risk models, as well as serum urea, for mortality by deriving their C-statistics, using receiver operator characteristic (ROC) curves. In general, a C-statistic value above 0.70 has acceptable discriminatory capacity. The C-statistics were compared to each other using a non-parametric test developed by DeLong et al. [8].

Survival for risk score categories for each scoring system was compared with Kaplan-Meier curves and the log-rank statistic.

To evaluate the ability of each scoring system to identify patients at risk of early (1-year) mortality following ICD implant, the sensitivity, specificity, positive predictive value (PPV) and negative predictive value (NPV) were calculated for each scoring system and urea.

Cox proportional hazards regression modelling was used to evaluate the relationship between each scoring system and the occurrence of appropriate and inappropriate ICD therapy during follow-up.

SPSS (version 21.0, SPSS Inc., Chicago, Illinois, USA) was used for the statistical analysis. The areas under the ROC curve for clinical event models were compared using MedCalc (version 15.8, MedCalc Software, Mariakerke, Belgium). A bilateral value of *P* < 0.05 was considered statistically significant.

## Results

### Baseline characteristics

The study cohort was composed of 406 patients (Table 2). The most common underlying aetiology was coronary artery disease (70.2%, *n* = 285) and the majority were primary prevention implants (58.4%, *n* = 237).

**Table 2:** Baseline characteristics of the study population. Values are median (interquartile range) or *n* (%)

Patient characteristics	(*n* = 406)
Patient demographics	
Age (years)	70 (60–76)
Male	322 (79.3)
Medical history	
Hypertension	236 (58.1)
Diabetes mellitus	99 (24.6)
Coronary artery Disease	285 (70.2)
Peripheral vascular disease	98 (24.1)
Previous PCI	153 (37.3)
Previous CABG	112 (27.6)
LVEF (%)	29 (23–38)
Cardiomyopathy	
Ischaemic	267 (65.8)
Non-ischaemic	139 (34.2)
Pre-implantation ECG	
QRS duration (ms)	130 (108–156)
Sinus	317 (78.1)
Atrial fibrillation/flutter	79 (19.4)
Heart block/paced	10 (2.5)
Pre-implantation blood results	
Urea (mmol/L)	7 (5.5–9.4)
eGFR (mLmin)	65 (50–81)
Haemoglobin level (g/dL)	12.9 (11.6–14.1)
Pre-implantation NYHA functional status	
I	139 (34.2)
II	130 (32)
III	129 (31.8)
IV	8 (2)
Type of device implanted	
Dual chamber ICD	124 (30.5)
CRT-D	183 (45.1)
Subcutaneous ICD	4 (1.0)
Indication for device implantation	
Primary prevention	237 (58.4)

*CABG* coronary artery bypass graft, *ICD* implantable cardioverter-defibrillator, *LVEF* left ventricular ejection fraction, *CRT-D* cardiac resynchronisation therapy-defibrillator, *PCI* percutaneous coronary intervention

### Predictors of mortality

During a mean follow-up of 936 ± 560 days, 96 patients died. In univariate Cox regression analyses, the absolute score of each scoring system was significantly associated with survival, with higher scores associated with worse survival (*P* < 0.0001 for all scoring systems) (Table 3). In addition, apart from AF, all risk factors included in each scoring system were also significantly associated with mortality (Table 3).

**Table 3:** Univariate and multivariate analyses for mortality by scoring system and constituent clinical parameters

Scoring system/clinical parameter	Univariate analysis			Multivariate analysis		
	Hazard ratio	95% CI	*P* value	Hazard ratio	95% CI	*P* value
Bilchick scoring system	1.009	1.007–1.012	<0.0001	1.004	1.001–1.007	0.008
Age ≥75 years	2.041	1.365–3.052	0.001			
NYHA class >II	1.879	1.259–2.805	0.002			
Atrial fibrillation	0.980	0.644–1.490	0.924			
COPD	2.130	1.320–3.436	0.002			
Chronic kidney disease	3.867	2.534–5.900	<0.0001			
LVEF ≤20%	1.746	1.118–2.727	0.014			
Diabetes mellitus	2.703	1.804–4.050	<0.0001			
Goldenberg scoring system	1.567	1.352–1.818	<0.0001			
NYHA class >II	1.879	1.259–2.805	0.002			
Age >70 years	2.101	1.374–3.212	0.001			
Urea >9.28 mmol/L	5.033	3.282–7.721	<0.0001	2.123	1.238–3.643	0.006
QRS duration >120 ms	2.082	1.339–3.237	0.001			
Atrial fibrillation	0.980	0.644–1.490	0.924			
Kramer scoring system	2.249	1.880–2.691	<0.0001	1.351	1.026–1.779	0.032
Peripheral arterial disease	3.944	2.639–5.894	<0.0001	1.842	1.082–3.135	0.024
Age ≥70 years	2.017	1.315–3.095	0.001			
Creatinine ≥177 μmol/L	2.434	1.853–3.198	<0.0001			
LVEF ≤20%	1.746	1.118–2.727	0.014			
Parkash scoring system	1.719	1.370–2.146	<0.0001			
Age >80 years	1.763	1.029–3.021	0.039			
Atrial fibrillation	0.980	0.644–1.490	0.924			
Creatinine >159 μmol/L	4.963	3.067–8.031	<0.0001			
NYHA class >II	1.879	1.259–2.805	0.002			

*CI* confidence interval, *COPD* chronic obstructive pulmonary disease, *NYHA* New York Heart Association, *LVEF* left ventricular ejection fraction

In multivariate analysis, including both the four risk scores as well as their individual components, the only independent predictors of mortality were the Kramer scoring system (*P* = 0.032), the Bilchick scoring system (*P* = 0.008), serum urea >9.28 mmol/L (*P* = 0.006) and peripheral arterial disease (*P* = 0.024).

### Comparison of risk model discrimination

The calibration of the Goldenberg, Bilchick, Kramer and Parkash scoring systems and urea for prediction of death were excellent, as demonstrated by the results of the Hosmer-Lemeshow test (Table 4).

**Table 4:** Calibration and discrimination of the Goldenberg, Bilchick, Kramer and Parkash scoring systems and urea for predicting death at 1- and 3-years following ICD implantation

		1 year							3 years						
		All patients	Ischaemic CM	Non-ischaemic CM	Primary		Secondary		All patients	Ischaemic CM	Non-ischaemic CM	Primary		Secondary	
					Single/dual ICD	CRT-D	Single/dual ICD	CRT-D				Single/dual ICD	CRT-D	Single/dual ICD	CRT-D
Bilchick	AUC	0.699	0.672	0.731	0.756	0.798	0.635	0.906	0.759	0.735	0.778	0.802	0.744	0.719	0.908
	95% CI	0.652–0.743	0.553–0.790	0.535–0.926	0.613–0.900	0.653–0.944	0.463–0.807	0.761–1.000	0.715–0.800	0.661–0.809	0.655–0.901	0.696–0.908	0.644–0.844	0.586–0.851	0.810–1.000
	H-L test	0.99	0.92	0.22	0.60	0.36	0.55	0.91	0.81	0.60	0.90	0.45	0.97	0.32	0.77
Goldenberg	AUC	0.606	0.571	0.665	0.609	0.676	0.633	0.979	0.667	0.627	0.729	0.741	0.648	0.682	0.779
	95% CI	0.557–0.654	0.443–0.699	0.487–0.842	0.416–0.802	0.505–0.847	0.479–0.788	0.927–1.000	0.619–0.713	0.545–0.710	0.617–0.841	0.629–0.853	0.534–0.763	0.546–0.818	0.570–0.987
	H-L test	0.19	0.35	0.17	0.36	0.45	0.38	1.00	0.76	0.92	0.23	0.55	0.99	0.97	0.54
Kramer	AUC	0.755	0.727	0.792	0.828	0.804	0.678	0.943	0.755	0.721	0.789	0.846	0.723	0.703	0.776
	95% CI	0.710–0.796	0.616–0.839	0.605–0.979	0.682–0.974	0.663–0.944	0.512–0.843	0.847–1.000	0.710–0.796	0.642–0.801	0.645–0.934	0.747–0.945	0.606–0.840	0.569–0.838	0.529–1.000
	H-L test	0.76	0.47	0.91	0.62	0.81	0.84	0.72	0.60	0.31	0.18	0.66	0.43	0.63	0.56
Parkash	AUC	0.568	0.536	0.609	0.573	0.601	0.502	0.948	0.632	0.611	0.652	0.665	0.608	0.583	0.739
	95% CI	0.518–0.617	0.411–0.662	0.355–0.863	0.384–0.762	0.409–0.794	0.319–0.686	0.864–1.000	0.583–0.679	0.524–0.699	0.504–0.799	0.532–0.798	0.485–0.730	0.424–0.743	0.523–0.954
	H-L test	0.30	0.48	0.11	0.32	0.64	0.42	1.00	0.37	0.72	0.18	0.83	0.86	0.50	0.48
Urea	AUC	0.711	0.695	0.699	0.820	0.830	0.614	0.958	0.703	0.697	0.666	0.725	0.802	0.635	0.710
	95% CI	0.664–0.755	0.578–0.812	0.457–0.940	0.689–0.950	0.731–0.930	0.448–0.780	0.875–1.000	0.656–0.747	0.612–0.782	0.507–0.826	0.603–0.847	0.693–0.910	0.491–0.778	0.427–0.992
	H-L test	0.80	0.77	0.45	0.44	0.34	0.83	0.54	0.20	0.53	0.06	0.32	0.42	0.56	0.44

*AUC* area under curve, *CI* confidence interval, *CM* cardiomyopathy, *CRT-D* cardiac resynchronisation therapy-defibrillator, *H-L* Hosmer-Lemeshow goodness-of-fit test, *ICD* implantable cardioverter-defibrillator

By calculating the area under the ROC curve, we evaluated the accuracy of each scoring system and urea to predict 1- and 3-year mortality (Table 4). The C-statistic values for the Kramer score (AUC 0.76–0.77), the Bilchick score (AUC 0.70–0.76) and urea (AUC 0.71–0.70) were consistently above 0.7, suggesting good discrimination. In contrast, the values for the Parkash and Goldenberg models were consistently below 0.7.

These findings were broadly consistent across the subgroups of primary and secondary prevention devices, as well as single/dual chamber ICDs and CRT-Ds (Table 4). However, the C-statistic values tended to be higher in the CRT-D subgroups compared to the single/dual chamber ICDs. Additionally, while there was a trend towards the C-statistic values being higher in the non-ischaemic cardiomyopathy (NICM) subgroup compared to those with ischaemic cardiomyopathy, the model calibration was generally better in those with ischaemic cardiomyopathy.

We compared the discriminative capacity of the four scoring system and urea to predict 1- and 3-year mortality. The C-statistics for the Kramer and Bilchick scores were significantly higher than either the Parkash or Goldenberg scores at both time points (*P* < 0.05) (Table 5). However, neither had significantly better discriminative capacity than urea for the prediction of 1- or 3-year mortality (Table 5).

**Table 5:** Comparison of C-statistics of the Bilchick, Goldenberg, Kramer and Parkash scoring systems and urea for mortality at 1- and 3-years

Comparison	Death at 1 year		Death at 3 years	
	*z*	*P* value	*z*	*P* value
Urea vs. Goldenberg	2.688	<0.01	1.133	0.26
Urea vs. Bilchick	0.324	0.75	1.883	0.06
Urea vs. Kramer	1.302	0.19	1.421	0.16
Urea vs. Parkash	3.027	<0.01	1.769	0.08
Goldenberg vs. Bilhick	2.566	0.01	3.708	<0.001
Goldenberg vs. Kramer	3.678	<0.001	2.864	<0.01
Goldenberg vs. Parkash	1.170	0.24	1.464	0.14
Bilchick vs. Kramer	1.439	0.15	0.131	0.90
Bilchick vs. Parkash	3.369	<0.001	4.107	0.0001
Kramer vs. Parkash	4.040	0.0001	3.472	<0.001

The null-hypothesis *z*-test result is shown for the comparisons of the C-statistic for the four scoring systems and urea, and respective *P* value, obtained by the DeLong non-parametric method

### Identifying patients at high risk of early mortality

Using the published cut-off values for each scoring system the proportion of ICD recipients in the high-risk groups were 5.9, 34.7, 7.4 and 21.4% for the Bilchick, Goldenberg, Kramer and Parkash scoring systems, respectively (Table 6). For urea (cut-off of >9.28 mM), the proportion was 25.1%. 1-year mortality in these five high-risk groups ranged from 11.7% (Goldenberg) to 40% (Kramer).

**Table 6:** Predictive accuracy of the Bilchick, Goldenberg, Kramer and Parkash scoring systems and urea for the prediction of 1-year mortality post-ICD implantation in 406 new ICD implants

Scoring system	Number in high-risk group	1-year mortality in high-risk group *n* (%)	1-year mortality prediction			
			Sensitivity	Specificity	PPV	NPV
			%	%	%	%
			(95% CI)	(95% CI)	(95% CI)	(95% CI)
Bilchick	24	7 (29.2)	21.2	95.4	29.2	93.2
			(9.0–38.9)	(92.8–97.3)	(12.6–51.1)	(90.2–95.5)
Goldenberg	141	16 (11.7)	48.5	66.5	11.4	93.6
			(30.8–66.5)	(61.5–71.3)	(6.6–17.8)	(89.9–66.2)
Kramer	30	12 (40.0)	36.4	9 5.2	40.0	94.4
			(20.4–54.9)	(92.5–97.1)	(22.7–59.4)	(91.6–96.5)
Parkash	87	11 (12.6)	33.3	79.6	12.6	93.1
			(18.0–51.8)	(75.2–83.6)	(6.5–21.5)	(89.7–95.6)
Urea	102	19 (18.6)	57.6	77.8	18.6	95.4
			(39.2–74.5)	(73.2–81.9)	(11.6–27.6)	(92.4–97.5)

*CI* confidence interval, *NPV* negative predictive value, *PPV* positive predictive value

**Fig. 1:**
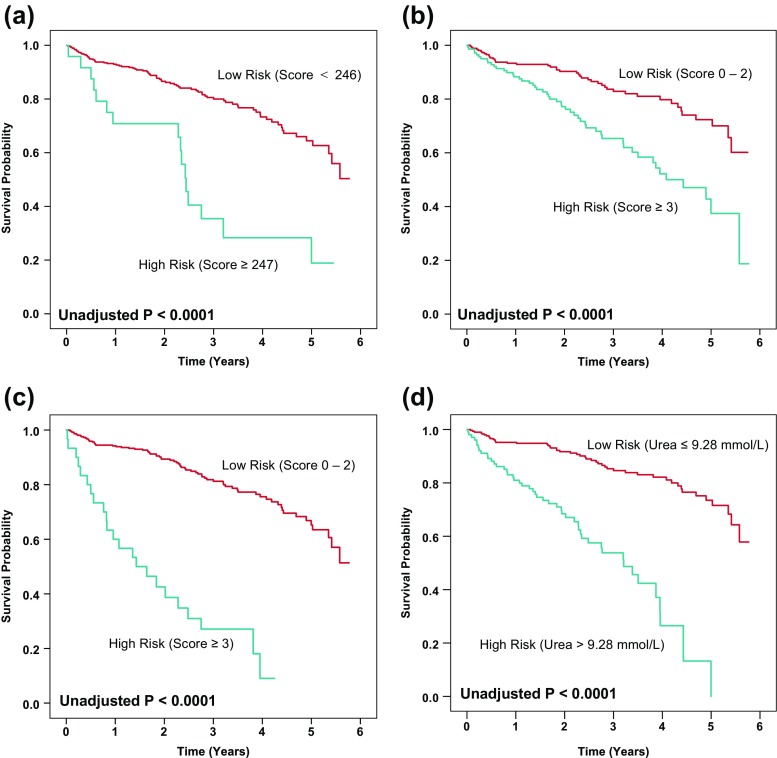
**a–d** Kaplan-Meier survival curves for survival following ICD implantation in different prognostic groups according to the **a** Bilchick, **b** Goldenberg, **c** Kramer scoring systems and **d** urea

For each of the four scoring system and urea, Kaplan-Meier survival analyses demonstrated significantly worse survival in high- compared to low-risk patients (*P* < 0.0001 for each analysis) (Fig. 1).

Overall, in our cohort, 1-year mortality was 8.1% (*n* = 33). Of these 33 patients, 8 (24.2%) received documented appropriate ICD therapy (ATP or shocks) and 2 (6.1%) received inappropriate ICD therapy prior to death. The scoring system that identified the largest proportion of these 33 patients was urea (*n* = 19, sensitivity 57.6%).

The sensitivity, specificity, PPV and NPV for each of the scoring systems and urea to predict 1-year mortality are shown in Table 6.

### Relationship of scoring systems to device therapy

During follow-up, 106 (26.1%) patients experienced appropriate device therapy (36 atp, 30 shock and 40 atp followed by shock) and 38 (9.4%) patients inappropriate therapy (18 atp, 12 shock and 8 atp followed by shock). In univariate cox regression analyses, only the Kramer scoring system was associated with the occurrence of appropriate device therapy (hazard ratio 3.15, *P* = 0.003) (Table 7). None of the scoring systems were associated with the occurrence of inappropriate therapy.

**Table 7:** Univariate and multivariate analyses for appropriate and inappropriate ICD therapies by scoring system or urea level

Scoring system/clinical parameter	Univariate analysis		
	Hazard ratio	95% CI	*P* value
Appropriate ICD therapy (shocks or atp)			
Bilchick scoring system	0.954	0.297–3.061	0.94
Goldenberg scoring system	1.230	0.702–2.154	0.47
Kramer scoring system	3.154	1.470–6.767	0.003
Parkash scoring system	1.513	0.830–2.761	0.18
Urea >9.28 mmol/L	1.141	0.590–2.204	0.70
Inappropriate ICD therapy (shocks or atp)			
Bilchick scoring system	0.046	0–260.063	0.48
Goldenberg scoring system	1.780	0.723–4.383	0.21
Kramer scoring system	1.865	0.430–8.088	0.41
Parkash scoring system	1.744	0.662–4.589	0.26
Urea >9.28 mmol/L	1.226	0.440–3.412	0.70

*CI* confidence interval

## Discussion

There are two main findings of this study. First, using published scoring systems, a significant proportion of current ICD recipients—between 6 and 35% in our cohort—are at high risk of early mortality following device implantation. Second, although all of the published scoring systems we evaluated predicted post-implant mortality, none significantly outperformed serum urea in terms of discrimination.

ICD therapy significantly improves survival in the low-LVEF patient population by the successful termination of ventricular arrhythmias that underlie preventable SCD. However, it has no impact on the risk of non-SCD. On the basis of results from multiple large controlled randomised (RCTs) trials, ICD therapy is targeted at patients at highest SCD risk. However, its clinical effectiveness is critically dependent not only on the risk of SCD but also on the risk of non-SCD [5, 9].

Using a simplified version of the Seattle Heart Failure Model, the SCD-HeFT investigators created a risk prediction model to divide the 2487 study patients into quintiles of increasing predicted baseline mortality risk [9]. Although in the overall study cohort ICD therapy improved survival, patients in the highest risk quintile of predicted mortality did not benefit from a device (relative risk for all-cause mortality 0.98, *P* = 0.89). There were similar findings in an analysis of the 1232 patients enrolled in MADIT-II, where again patients at highest pre-implant mortality risk failed to gain benefit from their ICD despite mortality benefit in the total study population [5].

Furthermore, it is possible that in some cases, ICD therapy may actually increase the risk of non-SCD. The occurrence of ICD shock therapy has been associated with worsening heart failure status and an excess mortality [10]. In addition, unnecessary right ventricular pacing may also worsen LVEF and increase mortality.

Although it is clear that some potential ICD recipients may be too sick to gain meaningful benefit from ICD therapy, it is unclear how best to accurately and reproducibly identify these patients prior to device implantation. The current guidance is limited to suggesting that ICD therapy is not indicated in patients with advanced heart failure, defined as New York Heart Association (NYHA) functional class IV, or in patients who do not have a reasonable expectation of survival with an acceptable functional status for at least a year [11]. However, there is no provision of how best to risk stratify patients in accordance with this guidance, making clinical interpretation difficult. Moreover, NHYA class is a relatively inaccurate prognostic variable, whose classification is often subjective.

A variety of alternative strategies to identify potential ICD recipients with an elevated non-SCD risk have been proposed. While early studies evaluated the use of individual risk markers, such as renal function and age, recent investigators have developed more complex risk scores in an attempt to improve prediction [4–7]. These different approaches reflect the observation that in the low-LVEF population, the main contributor to non-SCD is pump failure, though non-cardiac mortality may also play an important role in patients with significant comorbidity.

Our finding that urea, a measure of renal function, is a powerful marker of increased mortality following ICD implantation is consistent with published data. In a meta-analysis of patient-level data from 2867 patients enrolled in three RCTs of prophylactic ICD therapy, Pun et al. found that benefit from ICD therapy was strongly related to renal function, with impaired renal function at implant associated with a decrease in survival benefit from a device [12]. These findings have been reproduced by other investigators. These results emphasize the uncertain benefit of ICD therapy in patients with renal dysfunction and question the use of ICDs in this patient population.

Our data suggest that despite consensus guidelines stating that patients at increased short-term mortality risk should not receive ICD therapy, many such patients are still implanted. Using the four published scoring system in our cohort, 6 to 35% of implanted patients were identified to be at high risk of short-term mortality.

In our cohort, while all four scoring systems predicted survival, the Kramer and Bilchick scores had the best discriminative capacity, with C-statistics for both models consistently 0.7 or above for each of the two measured time points. However, despite this, none of the scoring systems outperformed serum urea, when evaluated using the area under the ROC curves. Furthermore, pre-implant serum urea, using the published cut-off of 9.28 mmol/L, identified the largest proportion (58%) of patients who died within 1 year of ICD implant.

When comparing predictive models, it is important to find a balance between mathematical accuracy and clinical applicability [13]. All of the proposed scoring systems used a minimum of four variables, with the Bilchick scoring system including seven variables and a nomogram to calculate the overall risk. In contrast, serum urea is universally available and simple to use.

Post hoc analysis of SCD-HeFT suggested a threshold of benefit may be present based on an annual mortality risk of 20–25%, with patients at greater annualised risk than this unlikely to benefit from an ICD [9]. Interestingly, 1-year mortality in the Kramer model (40%), Bilchick model (29%) and urea (19%) were all around this level. This supports the possibility that these models not only identify patients at high risk of mortality but also a group that may not benefit from ICD therapy.

The issue of identifying patients who fulfill current international guidelines but are unlikely to gain significant survival benefit from ICD therapy due to their high non-SCD risk is an important one. ICDs continue to be an expensive technology, and avoiding implanting patients who are unlikely to gain survival benefit is likely to improve clinical and cost-effectiveness. Furthermore, avoiding implanting unnecessary ICDs would prevent exposing patients with advanced cardiac and non-cardiac disease to the risks and potential complications of a high-energy device.

## Limitations

Our study has several potential limitations. First, it is a single-centre retrospective analysis and at risk of the inherent bias of this type of study. Although we analysed patients’ electronic records in detail, it is possible that important clinical variables used in the scoring systems, such as the presence or absence of PAD, were not recorded adequately. This may have resulted in a miscalculation of patients’ individual risk scores and impacted on our results.

Second, although we included data on 406 patients with 96 deaths, our analysis is relatively small by the standards of previous studies in this area.

Third, in our analysis, we included an unselected population of primary and secondary prevention patients, patients with CRT and non-CRT ICDs and patients with both ischaemic cardiomyopathy and NICM. The rationale for this was that the issue of identifying patients too sick to benefit from ICD therapy is important in all potential ICD recipients, irrespective of their ICD indication or the aetiology of their cardiac disease. However, some of the prediction models we evaluated were developed in purely primary prevention populations, or patients with only single/dual chamber ICDs, which may reduce their accuracy when evaluated in a mixed population. In addition, the recent publication of the DANISH study may impact on the guidelines for prophylactic ICD implantation in the NICM population [14]. For this reason, we have performed subgroup analyses based on indication, type of device and aetiology of cardiac disease.

Furthermore, when making decisions regarding complex device therapy for individual patients, it is important to balance the potential benefits against the risks. The benefit from ICD therapy is likely to be influenced by the indication (primary vs. secondary prevention), as well as the concomitant use of CRT. Although the association between the risk factors/models and mortality was relatively consistent across the different patient groups (primary/secondary prevention and ICD/CRT-D recipients), the numbers in each group are relatively small, and these models should be used with caution in patients with secondary prevention indications or CRT devices.

Fourth, given the observational design of our study, it is not possible to establish cause of death, which may have influenced interpretation of our results. However, all of the scoring systems we evaluated were designed to predict all-cause mortality and in none of the studies was cause or mode of death given.

Lastly, it is an observational study, and therefore, it is not possible to say that patients who died did not have their life meaningfully prolonged by ICD therapy.

## Conclusion

Using published scoring systems, a significant proportion of current ICD recipients are at high risk of short-term mortality following device implantation. Although all of the four published scoring systems we evaluated predicted early mortality following ICD implantation, none outperformed serum urea. We advocate the use of urea as a simple, clinically applicable, risk marker to better identify patients at high risk of early mortality post-ICD implantation.
